# Development of a Novel Gas-Sensing Platform Based on a Network of Metal Oxide Nanowire Junctions Formed on a Suspended Carbon Nanomesh Backbone

**DOI:** 10.3390/s21134525

**Published:** 2021-07-01

**Authors:** Taejung Kim, Seungwook Lee, Wootaek Cho, Yeong-Min Kwon, Jeong-Min Baik, Heungjoo Shin

**Affiliations:** 1 Department of Mechanical Engineering, Ulsan National Institute of Science and Technology (UNIST), Ulsan 44919, Korea; lgktj0305@unist.ac.kr (T.K.); zhffk9@unist.ac.kr (S.L.); dalgoo13418@unist.ac.kr (W.C.); 2 Department of Materials Science and Engineering, Ulsan National Institute of Science and Technology (UNIST), Ulsan 44919, Korea; kwonym@unist.ac.kr; 3 School of Advanced Materials Science and Engineering, Sungkyunkwan University (SKKU), Suwon 16419, Korea; jbaik97@skku.edu

**Keywords:** gas sensor, metal oxide nanowire, nanowire junction networks, suspended architecture, carbon nanomesh, C-MEMS

## Abstract

Junction networks made of longitudinally connected metal oxide nanowires (MOx NWs) have been widely utilized in resistive-type gas sensors because the potential barrier at the NW junctions leads to improved gas sensing performances. However, conventional MOx–NW-based gas sensors exhibit limited gas access to the sensing sites and reduced utilization of the entire NW surfaces because the NW networks are grown on the substrate. This study presents a novel gas sensor platform facilitating the formation of ZnO NW junction networks in a suspended architecture by growing ZnO NWs radially on a suspended carbon mesh backbone consisting of sub-micrometer-sized wires. NW networks were densely formed in the lateral and longitudinal directions of the ZnO NWs, forming additional longitudinally connected junctions in the voids of the carbon mesh. Therefore, target gases could efficiently access the sensing sites, including the junctions and the entire surface of the ZnO NWs. Thus, the present sensor, based on a suspended network of longitudinally connected NW junctions, exhibited enhanced gas response, sensitivity, and lower limit of detection compared to sensors consisting of only laterally connected NWs. In addition, complete sensor structures consisting of a suspended carbon mesh backbone and ZnO NWs could be prepared using only batch fabrication processes such as carbon microelectromechanical systems and hydrothermal synthesis, allowing cost-effective sensor fabrication.

## 1. Introduction

In the past decades, gas sensors have been widely applied in various fields, such as production facilities, automotive industry, medical technology, environmental protection, and industrial safety [1,2,3,4,5]. Among the various types of gas sensors, those based on metal oxide (MOx) are actively investigated owing to their high sensitivity, fast and reliable response, as well as simple operating principles [6,7,8,9,10]. In particular, the investigation of various MOx nanostructures has enabled the development of high-performance gas sensors with improved sensitivity, stability, and detection range, benefiting from the strained surface lattice, prevalence of step, edge, corner, and terrace sites, as well as high surface-to-volume ratio of these materials [11,12,13]. Among the various MOx nanomaterials investigated, one-dimensional (1-D) nanostructures possess a very high surface-to-volume ratio and aspect ratio, making them suitable for applications in gas sensors, whose operation is based on surface reactions [14,15]. In addition, MOx nanowires (NWs) can be synthesized using relatively simple methods, such as hydrothermal growth. This versatile method facilitates the fabrication of gas sensors with densely aligned MOx NWs [16,17]. In general, MOx nanomaterials form core–shell structures. For example, in the case of *n*-type metal oxides, the oxygen adsorbed on the surface creates an electron depletion layer, resulting in an increased electrical resistivity. Exposure to oxidizing or reducing gases alters the concentration of oxygen pre-adsorbed on the surface of *n*-type MOx nanostructures and thus the thickness of the depletion layer; in turn, this affects the amount of current flowing through the MOx structure. Therefore, the target gas can be monitored simply by measuring the resistance change of the MOx nanostructures in the gas sensors [18,19]. Furthermore, the sensitivity of the sensor can be further improved when the MOx nanostructures are connected to form junctions [20]. When 1-D MOx nanomaterials are grown and aligned on the substrate, they can connect with each other to facilitate the formation of dense junctions. These junctions act as additional current paths, creating a potential barrier at junction points when exposed to oxidizing or reducing gases [21]. The height of this potential barrier also changes as the metal oxide reacts with oxidizing or reducing gases. Thus, the electrical resistance of the MOx NW networks is determined by changes in both the depletion layer thickness and the potential barrier height; the latter conductivity change mechanisms have been reported to be more sensitive [22]. Therefore, the formation of junction networks has been widely employed to facilitate the fabrication of MOx-based gas sensors. In addition, the formation of NW networks is more efficient for sensor manufacturing processes compared to the fabrication of single wire-based sensors, which requires complex and difficult alignment procedures.

Another approach to improve the performances of resistive-type gas sensors involves separating the 1-D sensing structures from the substrate to form a suspended architecture [23,24,25,26]. When the sensing materials are suspended at a fixed distance from the substrate, the target gas can transfer more efficiently to the sensing sites, which allows eliminating substrate effects such as heat loss as well as stagnant layer effects. This approach can enhance sensor characteristics such as response and recovery time, limit of detection (LOD), and sensitivity. However, the conventional fabrication methods of suspended sensor platforms require complex processes and/or expensive equipment.

**Figure 1:**
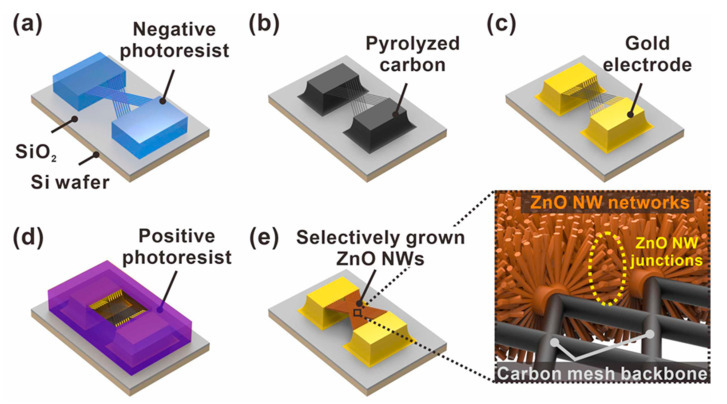
Schematic of the fabrication steps of suspended carbon nanomesh functionalized with ZnO NW networks. (**a**) Suspended polymer micromesh patterned by two-step photolithography; (**b**) conversion from polymer to carbon structures by pyrolysis; (**c**) gold electrode patterning; (**d**) selective exposure of suspended carbon mesh via photolithography; (**e**) selective integration of ZnO NW networks via ZnO seed layer deposition and hydrothermal growth processes (enlarged image: ZnO NW junctions with embedded carbon nanomesh backbone).

In this study, we present a novel gas sensor platform with improved sensing performance, achieved by forming ZnO NW junction networks in a suspended architecture via the radial growth of ZnO NWs onto the surface of a suspended carbon nanomesh, as shown in Figure 1. The suspended carbon mesh consists of sub-micrometer-sized carbon wires that intersect at a small, fixed interval. Thus, the ZnO NWs, grown radially from the surface of the carbon mesh wires facing each other, are connected longitudinally to form a dense junction network in the center of the void spaces of the mesh, as shown in Figure 1e. Our group had previously reported a simple method to fabricate monolithic mixed-scale carbon structures consisting of suspended 1-D carbon nanostructures supported by micrometer-sized carbon posts, using the carbon microelectromechanical systems (C-MEMS) process [27]. This approach enables the cost-effective wafer-level fabrication of complex carbon micro/nanostructures simply through the pyrolysis of photoresist structures pre-patterned by photolithography [28,29]. In addition, the pyrolysis is accompanied by a dramatic volume reduction, allowing the tailored fabrication of nanoscale carbon devices without requiring expensive and complex nanofabrication technologies [30,31,32]. In this study, suspended carbon mesh backbones were fabricated using C-MEMS processes, as described above. Then, a thin ZnO seed layer was selectively coated on the suspended mesh using photolithography and isotropic sputtering processes. Thus, ZnO NWs could be selectively grown on the mesh surfaces using a simple hydrothermal growth process, as shown in Figure 1d,e. Moreover, dense ZnO NW junction networks could be formed in a suspended architecture at a wafer level. As the integrated metal oxide nanowires are distributed circumferentially around the carbon mesh wires, the gas can efficiently access the sensing sites located at nanowire surfaces and junctions. To evaluate the effectiveness of the suspended network of metal oxide nanowire junctions, we fabricated various types of suspended ZnO NW-based sensors and compared their sensing properties. The suspended junction networks formed through the longitudinal connection of ZnO NWs exhibited enhanced response, LOD, and sensitivity compared with those measured using suspended and laterally connected ZnO NWs.

## 2. Materials and Methods

### 2.1. Fabrication of the Suspended Carbon Nanomesh Backbone

The fabrication steps of the present gas sensors are shown in detail in Figure S1. All processes were performed on a 6-inch Si wafer (*p*-type, boron-doped, 5–20 Ω·cm, thickness = 660–700 μm; LG Siltron Co., Ltd., Gumi-si, Korea). First, a 1 μm-thick SiO_2_ insulation layer was grown on the Si wafer by wet oxidation. Suspended polymer micromesh structures were fabricated via two successive photolithography steps. A negative photoresist (SU-8 2025, Microchem. Corp., Westborough, MA, USA) was spin-coated on the SiO_2_/Si substrate to a thickness of 25 μm and soft-baked at 95 °C for 8 min. This photoresist layer was exposed to UV light with a high dose (180 mJ·cm^−2^) from top to bottom to create post structures supporting a suspended micromesh. In the subsequent UV exposure, only the top portion of the photoresist layer was exposed to a low dose of UV light (15 mJ·cm^−2^), in order to form a suspended polymer micromesh. After post-exposure baking (95 °C for 7 min), the monolithic polymer structure consisting of the suspended polymer micromesh and the supporting post structures was obtained through a single development step. The polymer mesh structure was then carbonized into a carbon mesh by pyrolysis at 600 °C and 1 °C·min^−1^ in a vacuum furnace (Daemyoung, Ltd., Gwangmyeong, Korea). Because of the huge volume reduction during the pyrolysis process, the microscale polymer mesh shrank into a nanoscale carbon mesh structure. After pyrolysis, a 100 nm-thick Au layer was coated on the two carbon posts to compensate for the low electrical conductivity of the carbon posts pyrolyzed at low temperature. Thus, the electrical resistance between the two carbon posts mainly depended on the resistance of the ZnO NW networks. A positive photoresist (AZ 4330, AZ Electronic Materials, Somerville, NJ, USA) and e-beam evaporation (10 nm Cr/100 nm Au) were used for Au layer patterning.

### 2.2. ZnO Nanowire Growth on the Suspended Mesh Backbone

ZnO NWs were grown on the suspended carbon mesh via selective seed layer deposition followed by hydrothermal growth, as shown in Figure S1l–q. For the selective patterning of the ZnO seed layer, a positive photoresist (AZ 4330, AZ Electronic Materials, Somerville, NJ, USA) layer was spin-coated on the substrate. Then, only the top portion of the photoresist was exposed to a low dose of UV light. Thus, the unexposed photoresist layer under the suspended carbon nanomesh remained intact, and a 20 nm-thick ZnO seed layer could be selectively deposited onto the suspended carbon nanomesh by radio frequency (RF) sputtering and photoresist removal processes. After seed layer pattering, the ZnO NWs were hydrothermally grown using 10 mM zinc nitrate hexahydrate (Zn(NO_3_)_2_·6H_2_O, Sigma-Aldrich, St. Louis, MO, USA) and 10 mM hexamethylenetetramine ((CH_2_)_6_N_4_, Sigma-Aldrich, St. Louis, MO, USA) in an autoclave system.

### 2.3. ZnO Nanowire Characterization

The morphology and composition of the ZnO NWs were characterized using scanning electron microscopy (SEM; Quanta 200, FEI, Hillsboro, OR, USA), high-resolution X-ray diffraction (XRD; D8 Advance, Bruker, Billerica, MA, USA) using CuK_α_ radiation (average wavelength = 1.5418 Å), and high-resolution transmission electron microscopy (HRTEM; JEM-2100, JEOL, Ltd., Tokyo, Japan). A focused ion beam (FIB) milling machine (Helios 450HP, FEI, Hillsboro, OR, USA) was used for the preparation of the HRTEM samples.

### 2.4. Gas Sensing Tests

The sensing performances of the present sensor were evaluated for various gases such as NO_2_, SO_2_, CO, CH_4_, C_6_H_6_, and H_2_ using a gas chamber integrated with a heater (MPS-CHL, Nextron, Busan, Korea), as shown in Figure S2. Before the gas sensor tests, the gas-sensing chamber was prepared by several N_2_ purging and vacuum pumping cycles. The concentration of the target gas was controlled by mixing it with dry air as a carrier gas using a mass flow controller (GMC1200, Atovac, Yongin-si, Korea). The flow rate of dry air was fixed at 1000 sccm in all experiments. The change in the electrical resistance of the gas sensor was monitored using a source meter (Keithley 2450, Keithley Instruments, Inc., Cleveland, OH, USA), and the operating temperature was controlled using a ceramic heater stage installed inside the gas-sensing chamber. The experiments were carried out at atmospheric pressure. Gas response was represented as R_g_/R_a_ for oxidizing gas (NO_2_) and R_a_/R_g_ for reducing gases (SO_2_, CO, CH_4_, C_6_H_6_, and H_2_), where R_g_ and R_a_ represent the sensor resistance for target gas and dry air, respectively. The effect of humidity on gas detection was evaluated by mixing the target gas with wet air at controlled humidity (0–80% RH).

## 3. Results and Discussions

### 3.1. Morphology of the Gas Sensor Platform Based on the Suspended Network of ZnO NW Junctions

**Figure 2:**
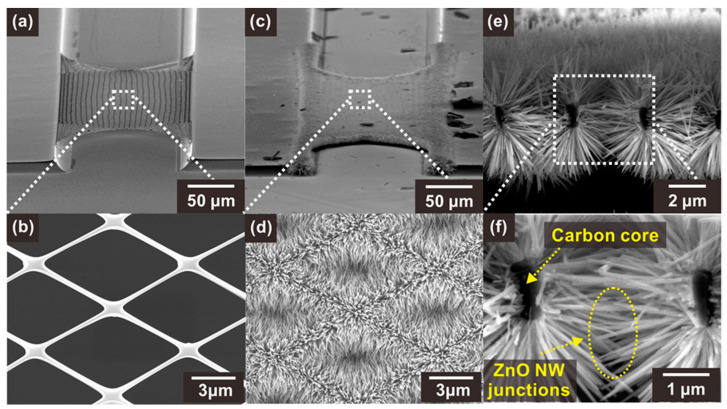
SEM images of (**a**,**b**) a bare suspended carbon nanomesh and (**c**,**d**) the same nanomesh functionalized with ZnO NWs. (**e**,**f**) SEM images of a section of carbon mesh showing a network of ZnO NW junctions.

The suspended carbon nanomesh backbone structures were fabricated using successive photolithography and pyrolysis processes, as shown in Figure 2a,b. The shape of the carbon nanomesh was maintained after pyrolysis, whereas the mesh size changed, as shown in Figure S3. As described in the experimental section, during the pyrolysis process, the polymer structure underwent a volume reduction of 40–90%, depending on its shape and size [27]. Thus, the width and thickness of the wires forming the mesh were reduced from 1 µm to 300 nm and from 4 µm to 0.6–1 µm, respectively. Similarly, the posts supporting the suspended structures shrank, and thus the distance between the two carbon posts increased from 120 to 140 µm. Nevertheless, the suspended carbon nanomesh did not show significant damage, because most of the volume reduction occurred before carbonization during the pyrolysis process [27].

The ZnO NWs were selectively integrated on the surface of the suspended carbon nanomesh by hydrothermal growth, which resulted in the formation of networks of NW junction. As shown in Figure 2d–f, ZnO NWs with a high aspect ratio (diameter ~60–90 nm, length ~2.5–3.5 µm) were densely grown and connected side by side on the carbon mesh. In addition, the ZnO NWs were distributed along the circumference of the carbon nanowires, facilitating the access of the target gas to the NW surface. The ZnO NWs grown on a carbon nanowire were long enough to connect with other NWs located across the void region of the mesh, forming dense NW junctions.

### 3.2. Microstructure and Composition of the ZnO Nanowires

The morphology and crystallinity of the ZnO NWs were analyzed through HRTEM and XRD, as shown in Figures S4 and S5, respectively. For the TEM analysis, the ZnO NWs were grown on a flat carbon pad under the same conditions used for the growth of the NWs on the suspended carbon mesh. The TEM images in Figure S4a,b show the ZnO NWs grown on a ZnO seed layer deposited onto carbon. Here, the seed layer acts as a nucleation site for the growth of the ZnO NWs. The corresponding selected-area electron diffraction (SAED) pattern shown in Figure S4c confirms the crystalline nature of the ZnO nanostructures, exhibiting unidirectional growth. The HRTEM image in Figure S4b displays the crystalline pattern of the grown ZnO NWs. In addition, the interplanar spacing of 0.26 nm shown in the inset of Figure S4b matches the spacing of the (002) crystal lattice of ZnO, confirming the growth of highly crystalline ZnO NWs along the *c*-axis.

The ZnO NW sample for the XRD analysis was prepared on a quartz wafer, to avoid the high-intensity Si peak. A carbon pad and ZnO NWs were also prepared using the same processes used for the suspended ZnO NWs. As shown in Figure S5, the XRD pattern displays a distinct (002) peak with full width at half maximum of 0.366°. The relatively higher intensity of the (002) peak, compared with that of the other lattice planes, indicates that the ZnO NWs were preferentially grown along the *c*-axis. The elemental compositions of ZnO NWs grown on a carbon nanomesh were quantitatively analyzed using energy-dispersive spectroscopy (EDS) measurements at two distinct spots, as shown in Figure S6. The EDS profile obtained from the spot corresponding to the ZnO NW surface shows the presence of zinc and oxygen in a 1:1 ratio, whereas no other elements were detected. This confirmed the pure ZnO composition of the as-grown NWs, with a negligible amount of impurities. The spectrum of the carbon core revealed a significantly higher carbon content.

### 3.3. Gas Sensor Characterization

The present gas-sensing mechanism is based on changes in electrical resistance, as the conductivity of the suspended ZnO NWs varies in response to the target gas concentration. Therefore, the electrical resistance of the carbon mesh should be high enough to ensure that the main current path is represented by the ZnO NW networks between the two carbon posts. The electrical conductivity of pyrolyzed carbon strongly depends on (and increases with) the pyrolysis temperature [33]. In this study, the carbon structure was pyrolyzed at 600 °C, resulting in a relatively low conductivity. Starting from this temperature, the rate of volume reduction during pyrolysis decreased, allowing efficient size control [27]. We evaluated the effect of the electrical resistance of the carbon structures on the total resistance of the suspended hybrid mesh by measuring the *I–V* curves shown in Figure S7. Although the deposition of a thin seed layer did not significantly affect the electrical resistance, the growth of the ZnO NW network reduced the total resistance by 50% at room temperature (Figure S7a). Moreover, at the high operating temperature (250 °C) of gas sensors, the total resistance of the hybrid mesh network was significantly reduced, and thus the effect of the carbon backbone on the total resistance became negligible (Figure S7b). In addition, the *I–V* curves show a good ohmic contact.

**Figure 3:**
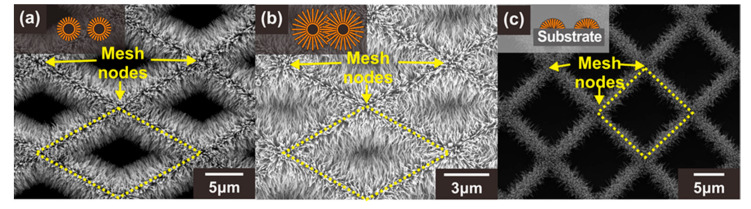
SEM images of three different types of ZnO NW junction networks: (**a**) Type A: ZnO NWs grown on the suspended carbon mesh with large voids, (**b**) Type B: ZnO NWs grown on the suspended carbon mesh with small voids leading to the formation of additional NW junctions, (**c**) Type C: ZnO NWs grown on the substrate. The insets show schematic illustrations of the cross section of each structure (orange: ZnO NWs, black: carbon mesh). Yellow dotted diamonds indicate the void region of the carbon mesh.

As explained in Section 1, when ZnO NWs are connected to each other forming network junctions, the depletion region or band bending increases significantly at the junctions with the exposure to oxidizing gases. This is because the electron density excited over the potential barrier exponentially decreases with the potential barrier [34]. Therefore, the potential barrier at the network junctions affects more sensitively the current flow along the MOX NW networks compared to the depletion layer thickness. In this study, three different types of ZnO NW junction networks were prepared to assess the effect of the suspended architecture and ZnO NW junctions on the gas detection properties. These sensor types were classified according to their carbon mesh void size and the architecture (suspended or substrate-bound): (A) ZnO NW junction networks grown on the suspended carbon mesh with large voids, (B) ZnO NW junction networks grown on the suspended carbon mesh with small voids, (C) ZnO NW junction networks grown on the substrate, as shown in Figure 3a–c, respectively. For a clear comparison, all types of sensors were prepared under the same ZnO NW growth conditions to obtain similar ZnO NW geometries. In addition, the size of the mesh void was designed differently to control the formation of the longitudinally connected NW junction networks (e.g., type A and B). When the size of the carbon mesh void area was large (type A), as shown in Figure 3a (side length of the unit diamond mesh ~14 µm), the ZnO NWs were connected transversely. For the carbon mesh with a small void region (side length of the unit diamond mesh ~5–6 µm), additional junctions were formed in the center of the void region of the carbon mesh (type B), as indicated by the yellow dotted diamonds in Figure 3b.

**Figure 4:**
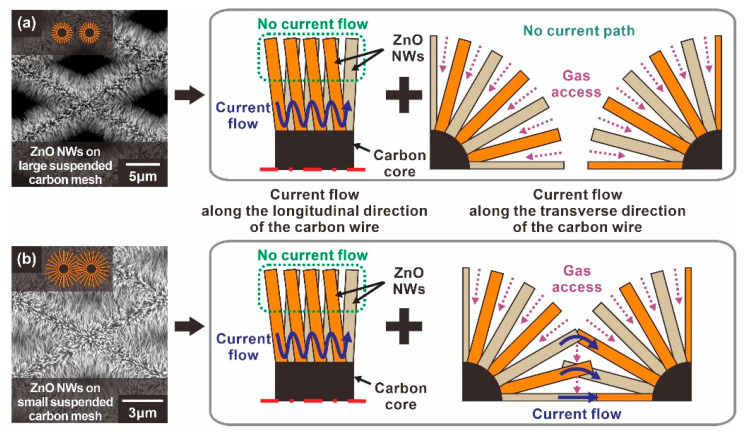
Schematic illustration of the current path of suspended ZnO NW networks according to the void size of the carbon mesh: (**a**) Type A: ZnO NWs grown on a suspended carbon nanomesh with voids too large to form longitudinally connected junctions, (**b**) Type B: ZnO NWs grown on a suspended carbon nanomesh with voids small enough to form longitudinally connected junctions.

For a type A sensor with ZnO NW networks mainly connected laterally as shown in Figure 3a, the ZnO NWs are tightly connected in the proximity of the carbon backbone due to the circumferential distribution of the ZnO NW networks. This tightly packed region serves as the main current path. On the other hand, the ZnO NW end regions, away from the carbon wire backbone, do not significantly participate in the current flow, as indicated by the dotted green lines in Figure 4a. Therefore, not all the depletion zone of the ZnO nanowire contributes to the overall change of electrical resistance of the ZnO NW networks. This type of current path also applies to the type B sensor based on the suspended carbon mesh with small voids, as shown in Figure 3b. In addition, additional current paths extend through longitudinally connected nanowires across the mesh void region, as shown in Figure 4b. In this case, most of the entire surface of these longitudinally connected ZnO NWs can be utilized as a current path. Moreover, enhanced gas sensing performances are expected, because the radially grown ZnO NW architecture allows the target gas to access the ZnO NW junctions more efficiently compared to the ZnO NW networks tightly connected in the lateral direction.

**Figure 5:**
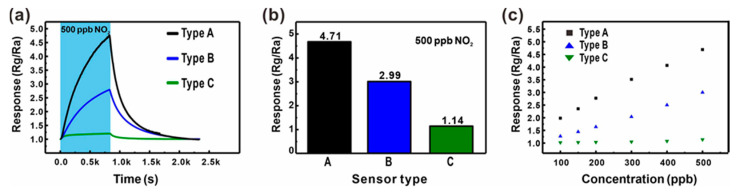
(**a**,**b**) Comparison of the gas sensing responses of three different types of ZnO NW networks (Type A: ZnO NW networks on a suspended carbon mesh with large voids (blue); Type B: ZnO NW networks on a suspend carbon mesh with small voids (black); Type C: ZnO NW networks on the substrate (green)) for 500 ppb NO_2_ mixed in dry air. (**c**) Comparison of the gas responses to various NO_2_ concentrations (100–500 ppb) mixed in dry air.

The effect of the additional junction networks on the gas detection properties was analyzed by comparing the gas sensing response to 500 ppb NO_2_ mixed in dry air measured using the three different types of ZnO NW junction networks, as shown in Figure 5a,b. The ZnO NW networks with additional longitudinally connected junctions (Type B: Black line and bar) exhibited a 57.5% higher response compared with the ZnO networks built on the large carbon mesh (Type A: Blue line and bar). A similar enhancement was also observed for other sensing properties such as sensitivity and LOD, as shown in Figure 5c. Accordingly, the LOD of ZnO NWs grown on the small carbon mesh was 30.6 ppb (*S*/*N* = 3), which was 36.1% better than that of ZnO NW networks without additional NW junctions. This is presumably due to the effect of additional junction-induced potential barriers. In addition, a gas can access most of the entire surface of the ZnO NWs in the mesh void region, in contrast to the limited access to the ZnO NWs laterally connected along the carbon wire backbone. However, both types of sensors exhibited similar responses and recovery times (Figure 5a), because they consisted of laterally connected ZnO NW junctions.

For the evaluation of the effect of the suspended architecture on the gas sensing performance of sensors based on ZnO NW junction networks, ZnO NW junction networks were prepared on the substrate, as shown in Figure 3c. For a clear comparison, the substrate-bound and suspended sensors were prepared under the same ZnO NW growth conditions. Due to the small size of the carbon mesh, accurate patterning of seed layer on the carbon structures was difficult. Instead, a ZnO seed layer was patterned without a carbon mesh backbone. Although the same photomask was used for mesh patterning, the resulting mesh shapes did not match (side length of the unit square mesh ~10–11 µm), as shown in Figure 3c, because of the volume change of the suspended carbon mesh during pyrolysis. As shown in Figure S3b and e, the two posts supporting the suspended mesh shrank during pyrolysis, and the mesh structure was extended towards the supporting posts. As a result, the shape of the mesh unit cell changed from square to diamond, as shown in Figure S3c,f, respectively. Therefore, the effect of the position of the longitudinally connected ZnO NW network on its gas sensing properties could not be analyzed. Instead, the analysis was performed on transversely connected ZnO NW-based sensors fabricated on the substrate. This is because the sensing characteristics of laterally connected junction network-based sensors are determined to a greater extent by the geometry of the laterally connected ZnO nanowires than by the shape and size of the carbon mesh backbone. While all three types of ZnO NW networks were grown under the same hydrothermal growth condition, the substrate-bound sensor exhibited shorter ZnO NWs (diameter ~40–60 nm, length ~10–15 µm) because of the limited mass transfer of the precursor to the seed layer on the substrate compared with the suspended sensor. The effect of the suspended architecture on the gas sensing performance of sensors based on the ZnO NW junction networks is shown in Figure 5a–c. The suspended sensor exhibited a higher response compared to the sensors placed on the substrate. ZnO NWs on the suspended mesh (blue figures) exhibited a 162% higher response for 500 ppb NO_2_ detection compared with the substrate-bound sensor (green figures). The sensitivity and LOD were also significantly enhanced by forming the ZnO NW junctions in a suspended architecture. This is because the suspended mesh facilitates a more effective gas transfer to the sensing sites without substrate effects [25,26,27]. In addition, the effect of the smaller NW length and density resulting from the limited mass transfer of the NW precursor to the substrate-bound mesh backbone should also be noted.

### 3.4. Gas Sensing Tests

**Figure 6:**
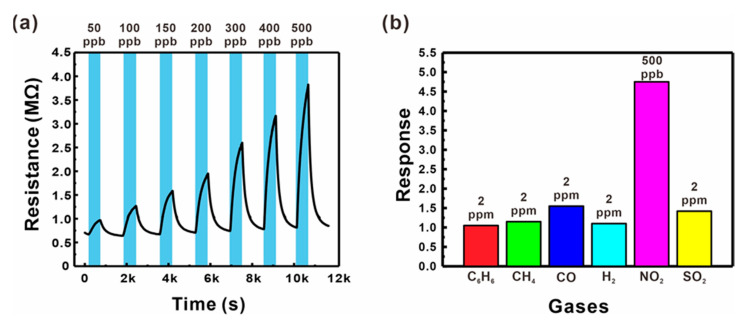
(**a**) Resistance changes of a suspended ZnO NW junction-based gas sensor (Type B) for various NO_2_ gas concentrations (50–500 ppb) mixed in dry air. (**b**) Responses of a suspended ZnO NW junction network-based sensor (Type B) to C_6_H_6_, CH_4_, CO, H_2_, NO_2_, and SO_2_ gases mixed in dry air.

The gas sensing performances of the sensor based on ZnO NWs grown on a suspended mesh with small voids (Type B, as shown in Figure 3b) were tested by measuring the changes in electrical resistance at a variety of operating temperature conditions (200–250 °C), as shown in Figures S8 and S9. The present sensor exhibited the highest response and fast reaction/recovery at 250 °C, and thus this temperature condition was selected for further sensor tests. The gas responses were measured for different NO_2_ concentrations (50–500 ppb), as shown in Figure 6a. The electrical resistance increased with the concentration of the oxidizing NO_2_ gas, reflecting the general behavior of *n*-type semiconducting materials. The present gas sensor exhibited a good gas response at very low concentrations (1.98 for 50 ppb), as well as a linear response with high sensitivity (0.92% ppb^−1^) up to 500 ppb (black squares in Figure 5c), resulting in a good LOD (30.6 ppb, *S*/*N* = 3). The sensor also exhibited changes in electrical resistance upon exposure to various reducing hazardous gases such as C_6_H_6_, CH_4_, CO, H_2_, and SO_2_, as shown in Figure 6b. Although the concentrations of the reducing gases were more than four times higher than that of NO_2_, the sensor responses to these gases were much lower compared to that measured for NO_2_, which can facilitate the selective detection of NO_2_. This is presumably because the potential barrier changes are more sensitive to the increased size of the depletion zone.

**Figure 7:**
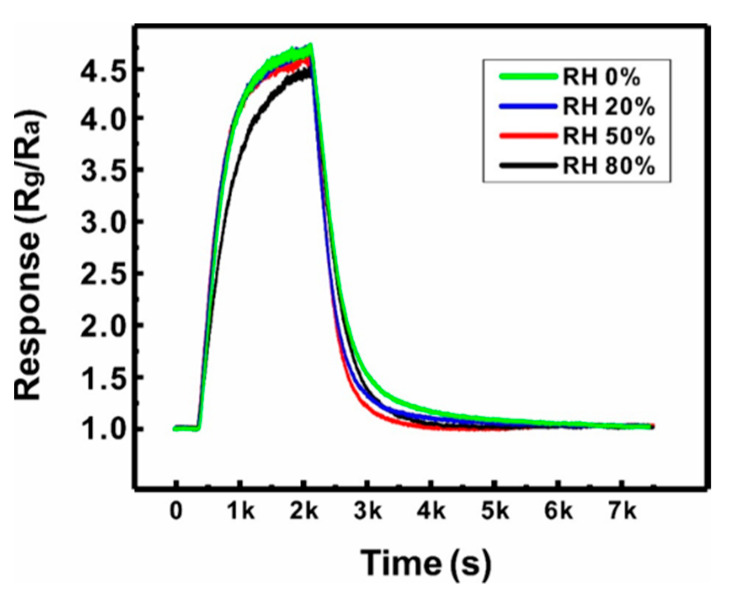
Effect of humidity (0, 20, 50, and 80% RH) on the gas response of a Type B sensor (500 ppb NO_2_, 250 °C).

The reliability of the sensor based on a suspended network of ZnO NW junctions was also assessed by evaluating the effect of humidity (0–80% RH) on the gas sensing performances (500 ppb NO_2_), as shown in Figure 7. The humidity in the sensing chamber was controlled by mixing the target gas with wet air at controlled humidity. The sensor exhibited negligible changes in gas response and response time until the humidity increased to 50% RH. When the humidity reached 80% RH, the response decreased by ~7%. This is because water molecule chemisorption occurs at the ZnO NW surface with the increase in humidity, forming adsorbed surface hydroxyl groups (OH*_(ad)_*), which hinders the surface reaction of NO_2_ [35]. However, this small change in response with respect to a wide range of humidity values (0–80% RH) indicates the reliability of the present sensor in practical applications.

## 4. Conclusions

In this study, we developed a novel gas sensor platform based on a suspended network of ZnO NW junctions formed on a carbon nanomesh backbone. The complex-shape ZnO NW networks could be selectively grown on the carbon mesh using a simple hydrothermal process; this is due to the suspended architecture and robust structure of the carbon mesh backbone. The void size of the carbon mesh was designed to be small enough to allow the formation of longitudinally connected junctions made of ZnO NWs grown from carbon meshes facing each other. The ZnO NWs were grown radially around the suspended carbon wire and connected in the center of the void regions of the mesh, resulting in efficient gas access to the sensing sites, including the longitudinally connected junctions and the entire NW surfaces. The enhanced gas access as well as the potential barrier at the junctions resulted in improved gas sensing performances such as response and sensitivity, compared to gas sensors based on either substrate-bound ZnO NW networks or suspended networks consisting of only laterally connected ZnO NW junctions. As shown in Table 1, the presented sensor showed comparable performances to those of other gas sensors based on various types of MOx NW junction networks, even though the presented ZnO MOx NW is a pristine semiconductor without any surface modification and heterogeneous material composition. In addition, the presented sensor fabrication method is based on a cost-effective wafer-level C-MEMS process. This process is compatible with various MOx nanostructures synthesis methods such as the vapor–liquid–solid (VLS) method under non-oxidizing conditions and the hydrothermal method, because pyrolyzed carbon has good chemical and thermal stability. Therefore, our sensor platform technology is easily accessible and compatible with various MOx NW synthesis methods and is therefore expected to contribute to enhancing the performances of gas sensors based on various types of MOx NWs. The suspended ZnO NW network-based sensor ensured reliable NO_2_ sensing performances even in humid environments. In addition, the mesh-like backbone of the present sensor can allow robust and reliable gas detection, because the gas sensor function can be maintained even if the sensor is subjected to slight structural damage; this is due to the redundant carbon wires forming the mesh backbone. In conclusion, this work provides a novel and cost-effective approach to facilitate the formation of junction networks of well-aligned metal oxide nanowires (widely used in nanowire-based gas sensors) in a suspended architecture. Because of its high temperature/chemical stability and robustness and the suspended architecture of the sensor backbone, the present gas sensor platform is expected to be applicable in gas-sensing devices based on various types of metal oxide nanowires. However, the limitation of the presented sensor platform should be noted. The current design of the sensor is not equipped with a low-power heater, which is required to heat ZnO NWs to the optimum operating temperature. This limitation can be overcome by utilizing the suspended carbon mesh backbone as a heater template, and therefore suspended MOx NW networks with a micro-heater can be implemented. In this heater-integrated sensor configuration, the carbon mesh backbone is selectively coated with a metal layer acting as a heater, and ZnO NWs are grown on the metal-coated mesh after insulation of the metal layer. This suspended heater-integrated MOx NW junction network-based sensor will be reported in the near future.

## Figures and Tables

**Table 1:** Summary of the sensing performances of gas sensors based on MOx NW junctions.

Configuration	MOx NW Growth Method	Type	Target Gas	Temperature (°C)	^1^ Concentration (ppm)	^2^ Response	^3^ LOD (ppb)	Reference
SnO_2_–ZnO hierarchical network	Hydrothermal	Substrate-bound	C_2_H_5_OH	400	25	3.0(R_g_/R_a_)	-	[36]
SnO_2_ NWs junction network	^4^ VLS	Substrate-bound	NO_2_	300	0.5	1.0(ΔR/R_a_)	-	[37]
ZnO NWs junction network	Hydrothermal	Substrate-bound	NO	70	50	0.147(ΔR/R_a_)	100	[38]
^5^ Au–ZnO/APTES NWsjunction network	^6^ Dielectrophoretic coating	Substrate-bound	NO_2_	~25	1	1.69(ΔR/R_a_)	-	[39]
ZnO NWs junction network	Hydrothermal	Suspended	NO_2_	250	0.5	4.71(R_g_/R_a_)	30.6	This work

^1^ Concentration: Lowest concentration of target gas measured in each study; ^2^ Response: Gas sensor response at ^1^ the lowest measurable concentration and at the optimal temperature; ^3^ LOD: Calculated limit of detection; ^4^ VLS: Vapor–Liquid–Solid method without an oxidation condition, ^5^ Au–ZnO/APTES: Au-doped ZnO NW with a (3-aminopropyl) triethoxysilane layer; ^6^ Dielectrophoretic coating: Aligning pre-grown ZnO NWs (in powder form) using dielectrophoretic force.

## Data Availability

The data presented in this study are available on request from the corresponding author.

## Supplementary Materials

The following are available online at https://www.mdpi.com/article/10.3390/s21134525/s1, Figure S1: Schematic fabrication steps of the suspended carbon nanomesh functionalized with ZnO NWs (PR: Photoresist), Figure S2: (a) Schematic of the gas sensing experiment setup. (b) Photograph of the-sensing chamber, Figure S3: SEM images of a (a–c) suspended polymer micromesh before pyrolysis and (d–f) the corresponding suspended carbon nanomesh after pyrolysis: (a,d) Bird-eye view. (b,c,e,f) Top view, Figure S4: TEM analysis results of ZnO–NWs grown on a carbon pad: (a) TEM image of the overall sample structure, (b) HRTEM image, and (c) corresponding diffraction pattern, Figure S5: XRD pattern of ZnO NWs grown on a pyrolyzed carbon thin film in the quartz substrate, Figure S6: EDS analysis of the suspended carbon nanomesh functionalized with ZnO NWs: Point chemical analysis spectrum from (a) ZnO NWs and (b) carbon nanomesh, Figure S7: I–V curves of suspended nanomesh structures (black line: Bare carbon nanomesh, red line: ZnO seed layer/carbon mesh, blue line: ZnO NWs/ZnO seed layer/carbon mesh) measured at (a) room temperature and (b) 250 °C, Figure S8: Gas sensing responses of a Type B sensor (ZnO NW junction networks grown on a suspended mesh with small voids) to 500 ppb NO_2_ at various operating temperature conditions (200–300 °C), Figure S9: (a) Gas response and (b) response (blue)/recovery(red) time for various operating temperature conditions corresponding to the results shown in Figure S8.
