# Supplementary material for: Development of a Novel Gas-Sensing Platform Based on a Network of Metal Oxide Nanowire Junctions Formed on a Suspended Carbon Nanomesh Backbone
Source: Sensors (Basel). 2021 Jul 1;21(13):4525. doi: 10.3390/s21134525 (PMC8272173; doi:10.3390/s21134525)
Supplement: Supplementary file 1 [file sensors-21-04525-s001.zip › sensors-1258410-supplementary.pdf]

Supplementary Material

Development of a Novel Gas-Sensing Platform Based on a Network of Metal Oxide Nanowire Junctions Formed on a Suspended Carbon Nanomesh Backbone

Taejung Kim [1,†], Seungwook Lee [1,†], Wootaek Cho [1], Yeong Min Kwon [2], Jeong Min Baik [3] and Heungjoo Shin [1,*]

[1] Department of Mechanical Engineering, Ulsan National Institute of Science and Technology (UNIST), Ulsan 44919, Korea; lgktj0305@unist.ac.kr (T.K.); zhffk9@unist.ac.kr (S.L.); dalgoo13418@unist.ac.kr (W.C.)
[2] Department of Materials Science and Engineering, Ulsan National Institute of Science and Technology (UNIST), Ulsan 44919, Korea; kwonym@unist.ac.kr
[3] School of Advanced Materials Science and Engineering, Sungkyunkwan University (SKKU), Suwon 16419, Korea; jbaik97@skku.edu
* Correspondence: hjshin@unist.ac.kr; Tel.: +82-52-217-2315
† These authors contributed equally to this work.

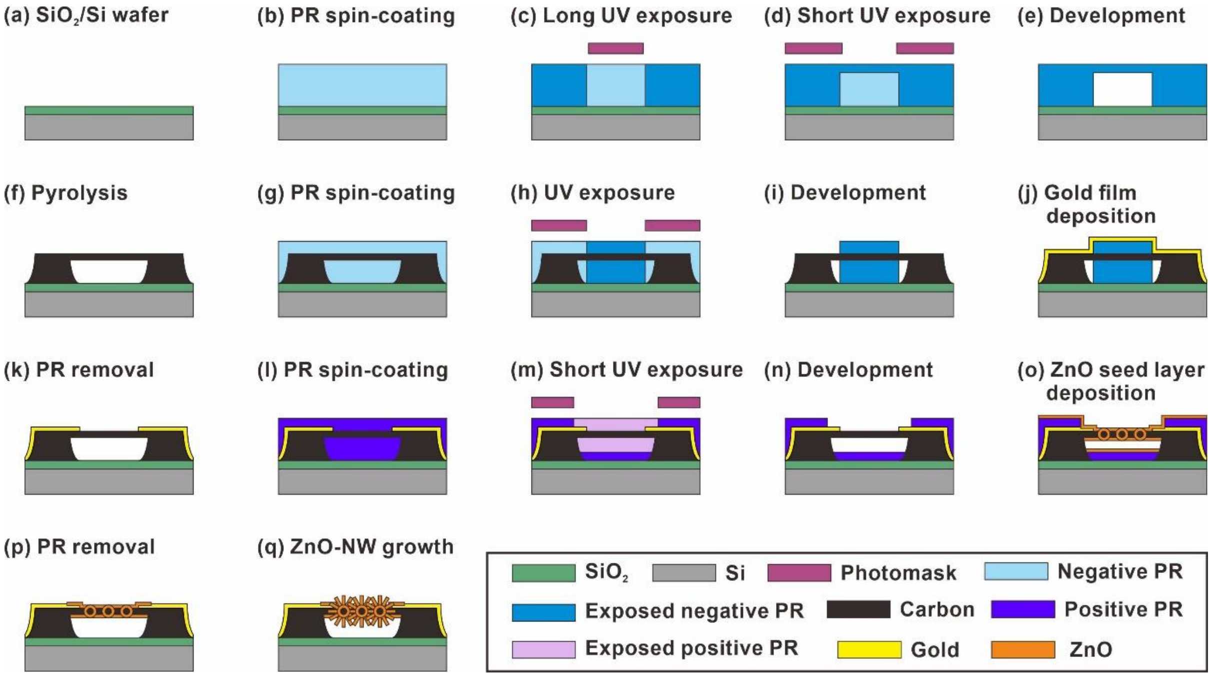

Figure S1. Schematic fabrication steps of suspended carbon nanomesh functionalized with ZnO NWs (PR: photoresist).

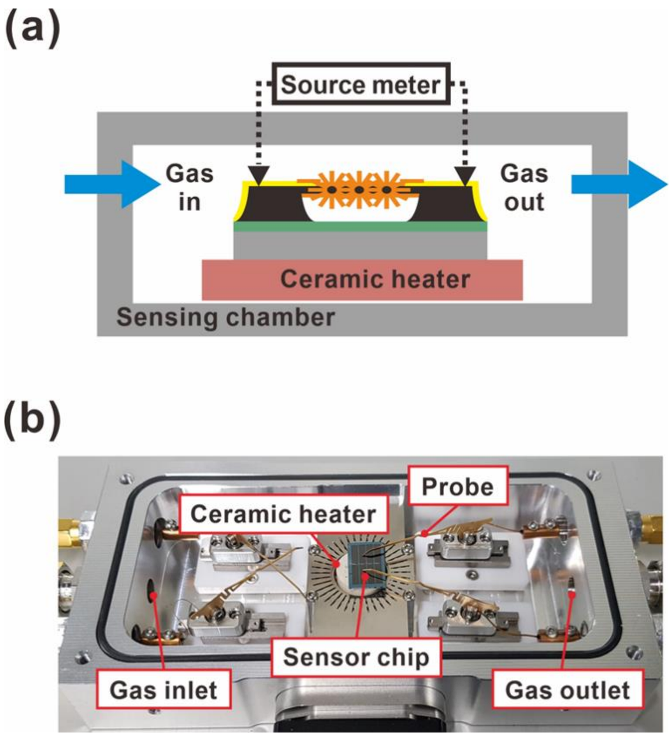

Figure S2. (a) Schematic of gas sensing experiment setup. (b) Photograph of the sensing chamber.

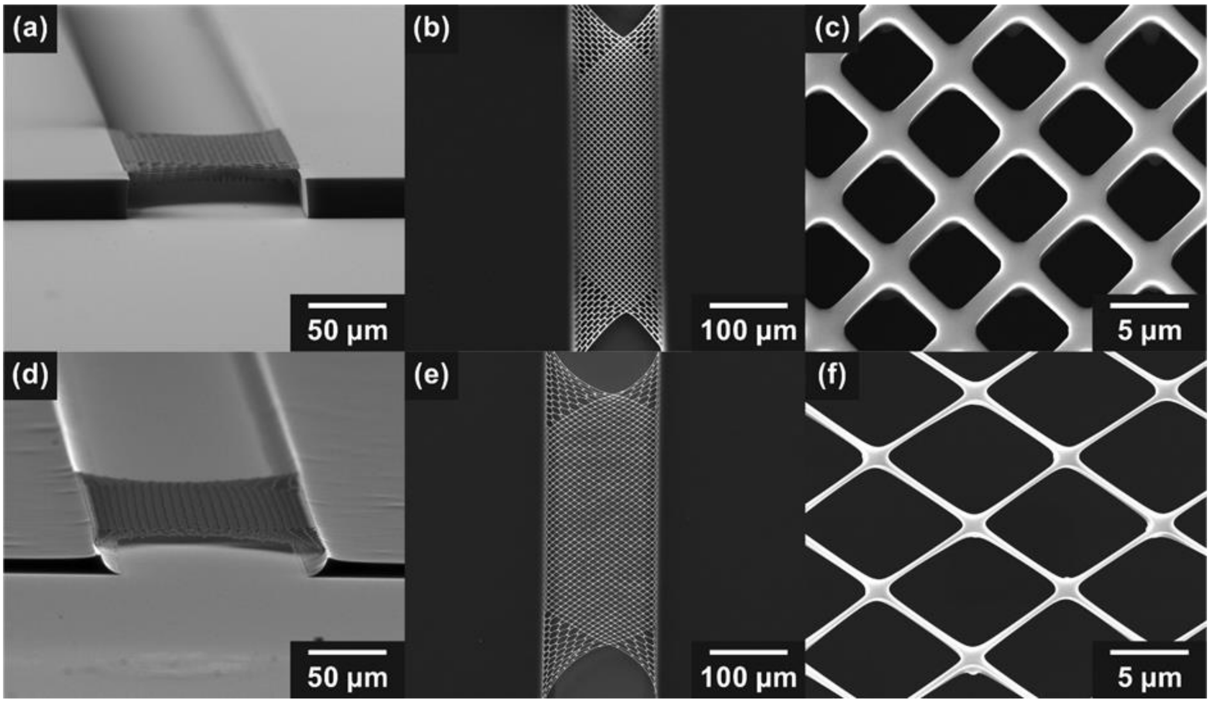

Figure S3. SEM images of (a-c) suspended polymer micromesh before pyrolysis and (d-f) corresponding suspended carbon nanomesh after pyrolysis: (a, d) Bird-eye view. (b, c, e, f) Top view.

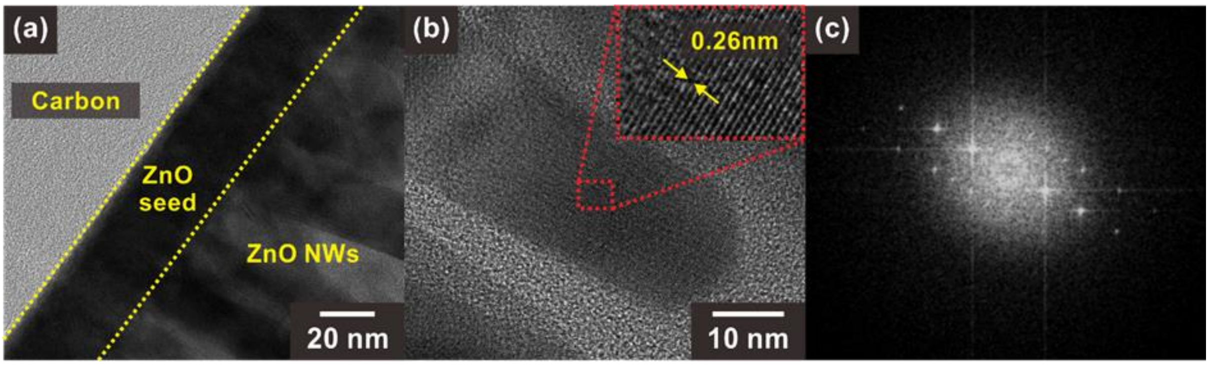

Figure S4. TEM analysis results of ZnO-NWs grown on a carbon pad: (a) TEM image of the overall sample structure, (b) HRTEM image, and (c) corresponding diffraction pattern.

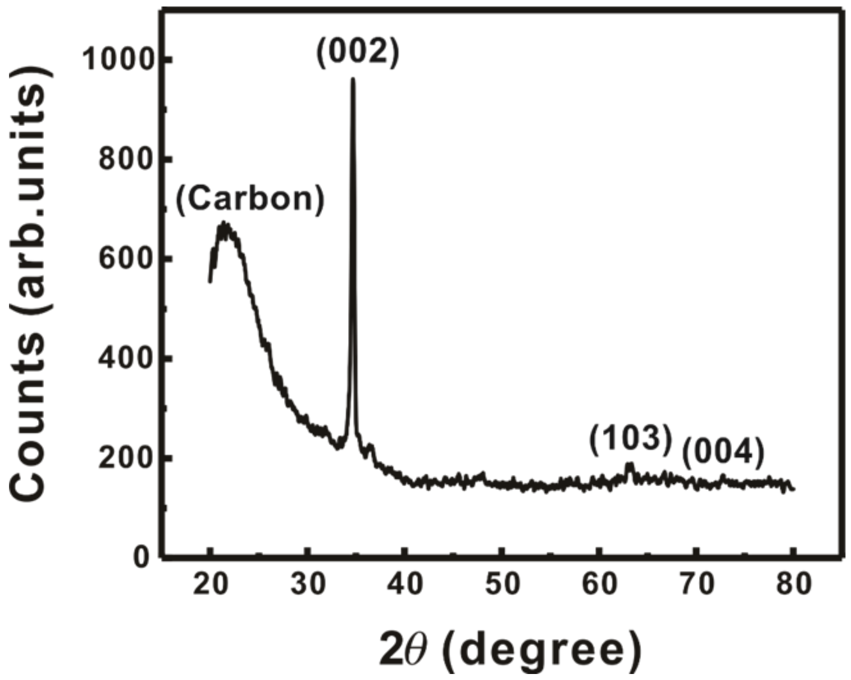

Figure S5. XRD pattern of ZnO NWs grown on a pyrolyzed carbon thin film in the quartz substrate.

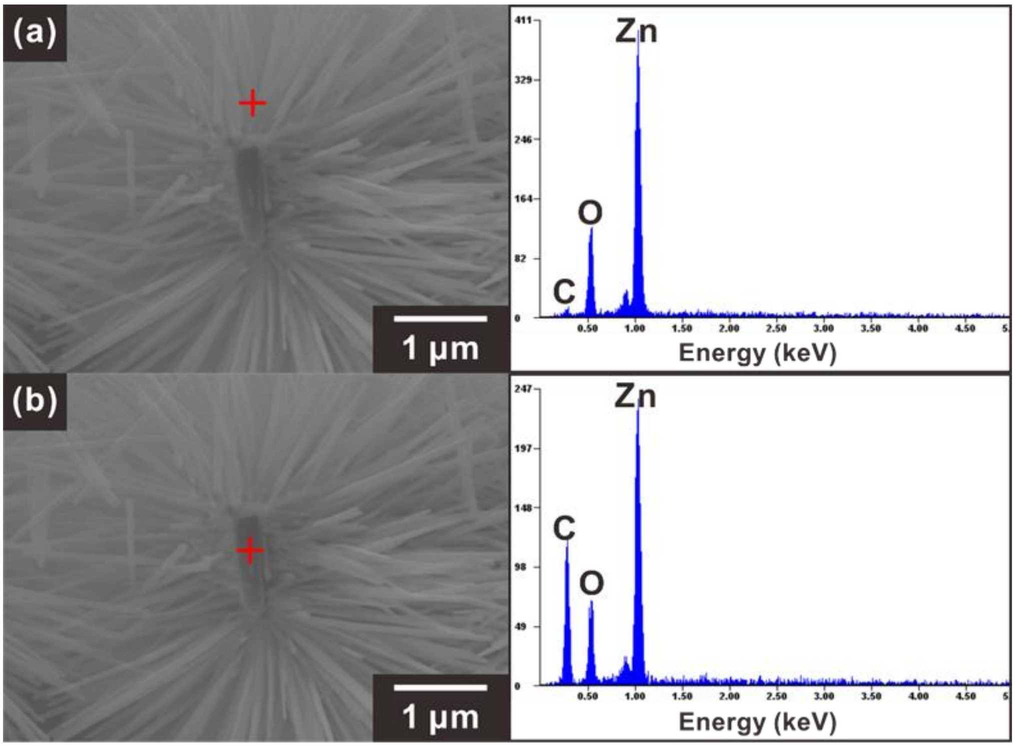

Figure S6. EDS analysis of the suspended carbon nanomesh functionalized with ZnO NWs: Point chemical analysis spectrum from (a) ZnO NWs and (b) carbon nanomesh.

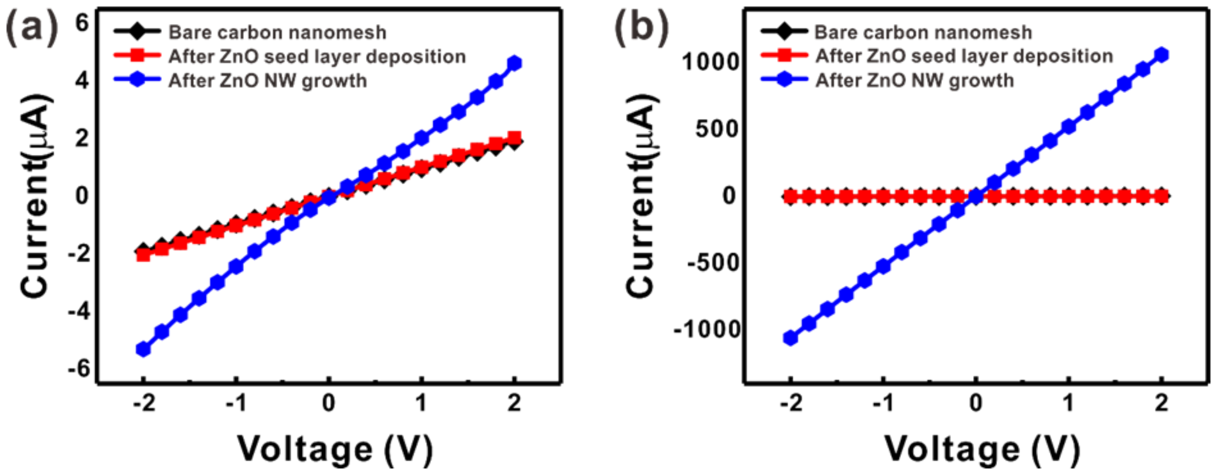

Figure S7. I-V curves of suspended nanomesh structures (black line: bare carbon nanomesh, red line: ZnO seed layer/carbon mesh, blue line: ZnO NWs/ZnO seed layer/carbon mesh) measured at (a) room temperature and (b) 250 °C.

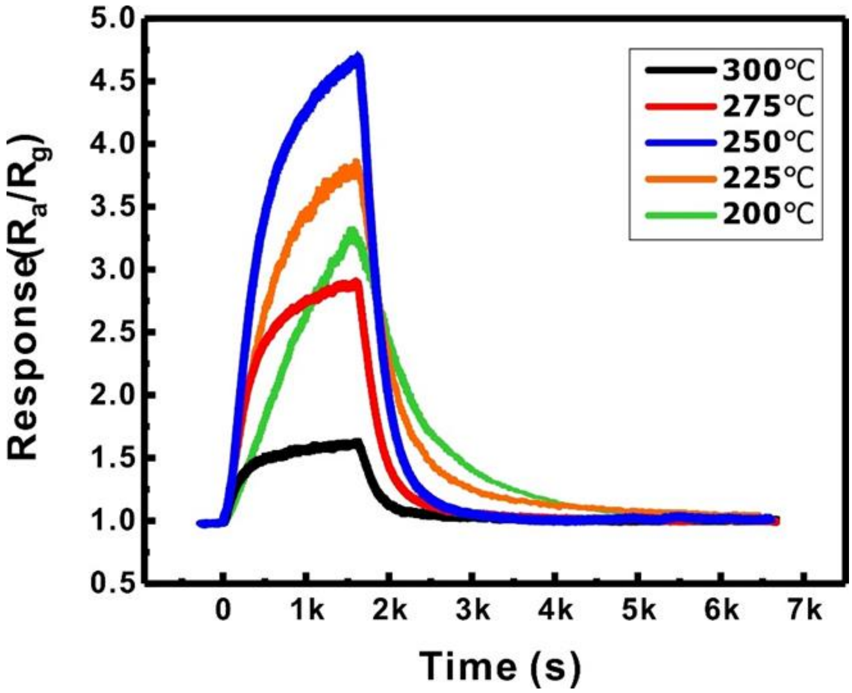

Figure S8. Gas sensing responses of a Type B sensor (ZnO NW junction networks grown on a suspended mesh with small voids) to 500 ppb NO_2 at various operating temperature conditions (200–300 ℃).

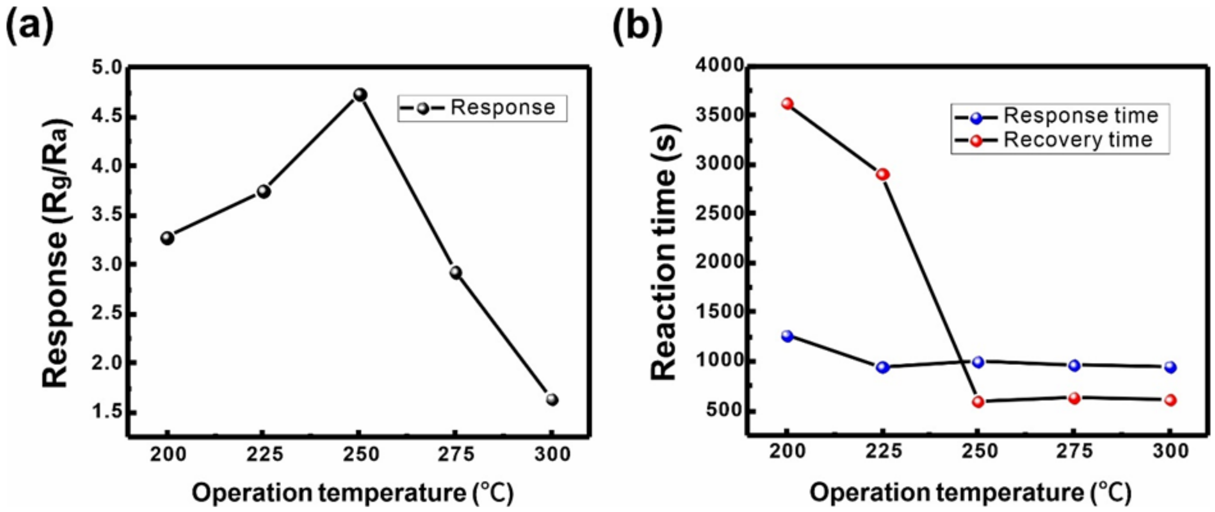

Figure S9. (a) Gas response and (b) response (blue)/recovery(red) time for various operating temperature conditions corresponding to the results shown in Figure S8.
